# Overcoming *Pseudomonas aeruginosa* in Chronic Suppurative Lung Disease: Prevalence, Treatment Challenges, and the Promise of Bacteriophage Therapy

**DOI:** 10.3390/antibiotics14050427

**Published:** 2025-04-23

**Authors:** Jagdev Singh, Melinda Solomon, Jonathan Iredell, Hiran Selvadurai

**Affiliations:** 1 Department of Respiratory Medicine, The Children’s Hospital at Westmead, Sydney, NSW 2145, Australia; hiran.selvadurai@health.nsw.gov.au; 2 Faculty of Medicine, University of Sydney, Sydney, NSW 2145, Australia; jonathan.iredell@sydney.edu.au; 3 Department of Respiratory Medicine, Hospital for Sick Children, Toronto, ON M5G 1X8, Canada; melinda.solomon@sickkids.ca; 4 Faculty of Medicine, University of Toronto, Toronto, ON M5S 1A1, Canada; 5 Westmead Institute of Medical Research, Sydney, NSW 2145, Australia

**Keywords:** *Pseudomonas aeruginosa*, bacteriophage, multi-drug resistance, cystic fibrosis, chronic suppurative lung disease

## Abstract

*Pseudomonas aeruginosa*, a multidrug-resistant pathogen, significantly impacts patients with chronic respiratory conditions like cystic fibrosis (CF) and non-CF chronic suppurative lung disease (CSLD), contributing to progressive lung damage and poor clinical outcomes. This bacterium thrives in the airway environments of individuals with impaired mucociliary clearance, leading to persistent infections and increased morbidity and mortality. Despite advancements in management of these conditions, treatment failure remains common, emphasising the need for alternative or adjunctive treatment strategies. Bacteriophage therapy, an emerging approach utilising viruses that specifically target bacteria, offers a potential solution to combat *P. aeruginosa* infections resistant to conventional antibiotics. This review examines the prevalence and disease burden of *P. aeruginosa* in CF and CSLD, explores the mechanisms behind antibiotic resistance, the promising role of bacteriophage therapy and clinical trials in this sphere.

## 1. Introduction

*Pseudomonas aeruginosa* is a gram-negative, opportunistic bacterium commonly associated with acute or chronic infections, including nosocomial infections, infections in immunocompromised individuals, and those with structural lung diseases. Notably, it is one of the ESKAPE pathogens (*Enterococcus faecium, Staphylococcus aureus, Klebsiella pneumoniae, Acinetobacter baumannii, P. aeruginosa,* and *Enterobacter* species) known for multidrug resistance (MDR) [1]. Approximately one-third of overall *P. aeruginosa* isolates demonstrate carbapenem resistance, with infections caused by extensively drug-resistant (XDR) strains linked to a 40% increase in mortality [1].

This opportunistic bacterium is commonly associated with chronic respiratory infections in patients with chronic airway inflammation, particularly those with cystic fibrosis (CF) or non-CF chronic suppurative lung disease (CSLD). These conditions are characterised by early and persistent airway inflammation, impaired mucociliary clearance, and frequent respiratory infections, which collectively contribute to progressive lung damage and poor clinical outcomes [2]. In these compromised hosts, a favourable ecological niche enables the bacterium to adapt, evolve and perpetuate itself, making eradication from the lungs a challenge [3].

Despite advances in antibiotic therapy, treatment failure and antibiotic resistance remains a significant challenge, leading to prolonged infections, hospital admissions, and a need for innovative approaches or adjuncts to antibiotic therapies [4,5,6]. Bacteriophage therapy, which uses viruses to specifically target and kill bacteria, is emerging as a potential solution to antibiotic limitations. This review explores the role of bacteriophage therapy in *P. aeruginosa* infections in CSLD. While global interest in bacteriophage therapy is growing, several critical knowledge gaps remain—particularly regarding its application in paediatric populations, its use in CSLD, optimal delivery methods for respiratory infections, and the dynamics of bacteriophage–antibiotic synergy. Furthermore, most of the available evidence arises from pre-clinical trials, compassionate access schemes or early-phase clinical trials, with a paucity of studies focused on children or chronic respiratory infections outside of CF.

Here, we summarise the burden and pathophysiology of *P. aeruginosa* in CSLD, examine the mechanisms by which bacteriophages can overcome bacterial resistance, evaluate current evidence from clinical trials and compassionate use programmes, and highlight future research and clinical application directions.

## 2. Prevalence and Disease Burden of *Pseudomonas aeruginosa* in CF Lungs

CF is an autosomal recessive genetic disorder caused by the absence, reduction, or impaired function of the cystic fibrosis transmembrane conductance regulator (CFTR) protein. The resultant dysfunction of the CFTR protein, a regulated anion channel located in the apical membrane of epithelial cells in organs such as the lungs, liver, pancreas, and gastrointestinal tract, leads to a multisystemic condition with significant effects [7]. In the lungs, CFTR dysfunction results in inflammation of the airways and the production of thick, sticky mucus, which leads to recurrent respiratory infections and lung damage. Based on registry reports, CF affects nearly 100,000 individuals worldwide; however, estimates suggest that the true prevalence, including unreported cases, may be closer to 160,000 individuals [7,8]. 

Over the past two decades, advancements in CF management and treatment have led to a decline in *P. aeruginosa* lung infections in individuals with CF. These improvements stem from improved infection control, enhanced surveillance, and a deeper understanding of CF’s natural course. Earlier diagnosis through neonatal screening, targeted treatments for respiratory infections and inflammation, optimised mucociliary clearance, and improved nutrition have all contributed to better outcomes. Comprehensive CF care delivered by experienced multidisciplinary teams has also played a key role, supporting adherence to evolving treatment strategies. While these changes preceded the introduction of CFTR modulator therapy, the addition of CFTR modulators holds promise for further improving outcomes by reducing inflammation and mucus secretion [9,10,11]. Infections caused by *P. aeruginosa* have decreased from approximately 60% to 30%, particularly in more recent birth cohorts [11,12,13]. Despite the decline in prevalence, *P. aeruginosa* infection continues to play a significant role in disease progression, with many individuals still facing chronic *P. aeruginosa* infection and a more rapid deterioration of lung function. In a French birth cohort study of children born after 2001, the median age at *P. aeruginosa* infection was five years, with one-quarter becoming chronically colonised by *P. aeruginosa* just before their 15th birthday [14]. Similarly, a study from the United States on children born after 2006 found that one in five children living with CF developed chronic *P. aeruginosa* infection by the age of 10 years [15]. More importantly, the annual decline in lung function following *P. aeruginosa* infection was about three to five times faster than in those without the infection [14]. Treatment of initial infection with *P. aeruginosa* tends to fail in up to 40% of patients treated with antibiotics, leading to chronic infection [16]. Chronic *P. aeruginosa* infection is a major contributor to recurrent pulmonary exacerbations, which are associated with increased morbidity and mortality, and is often the primary endpoint in clinical trials. About one in four patients with pulmonary exacerbations due to chronic *P. aeruginosa* infection fail to respond to treatment during a pulmonary exacerbation, resulting in lower lung function and further readmissions [17,18,19]. This failure rate is even higher in patients infected with drug-resistant strains of *P. aeruginosa*, which is concerning given the high levels of antibiotic resistance observed in this bacterium [17,20].

## 3. Prevalence and Disease Burden of *Pseudomonas aeruginosa* in Non-CF Chronic Suppurative Lung Disease

CSLD is an umbrella term encompassing a clinical spectrum ranging from protracted bacterial bronchitis to bronchiectasis. It is characterised by chronic or recurrent wet (or productive) cough and lower airway infections [21]. In children and adolescents, CSLD can also present with symptoms such as faltering growth, exertional dyspnoea, digital clubbing, and chest wall deformities [22]. Similar to CF, conditions leading to CSLD result in chronic airway inflammation, persistent infection, impaired mucociliary clearance, and progressive lung damage [21]. While similar in terms of symptoms, there is a significant equity gap compared to those with CF, primarily due to under recognition, diagnostic delays, and, consequently, delays in treating treatable traits. This leads to poorer lung function, less structured follow-ups, and overall suboptimal care compared to the well-established clinical pathways for CF [22].

CSLD includes a wide range of underlying conditions, such as immunodeficiency, primary ciliary dyskinesia, recurrent aspiration, inhaled foreign bodies, connective tissue diseases, chronic obstructive pulmonary disease (COPD), or inflammatory bowel disease [22].

Population-based studies conducted over a decade have shown an increasing prevalence of bronchiectasis, with rates between 350 and 566 per 100,000 women and 310 to 485 per 100,000 men. Studies in children have shown a prevalence of 180 per 100,000 children in New Zealand [23,24]. Similar increases in incidence and prevalence of CSLD and bronchiectasis have been described in the United States [25]. Among children, these conditions are particularly prevalent in socially disadvantaged populations, such as the Indigenous peoples of Canada, Australia, and New Zealand, with incidence rates as high as 735 per 100,000 in Central Australia [26].

Although the overall prevalence of *P. aeruginosa* in CSLD is generally lower than in CF (around 3%), studies focusing on bronchiectasis have reported *P. aeruginosa* isolation in one in five bronchoalveolar lavage samples [27,28]. Unsurprisingly, chronic infection with *P. aeruginosa* in CSLD is also associated with a rapid decline in lung function, more complications and an increased frequency of exacerbations [29,30]. Mucoid strains of *P. aeruginosa* account for half of the isolates in patients with bronchiectasis, which predicts a poorer prognosis [31]. The eradication rate of *P. aeruginosa* in patients with CSLD and bronchiectasis is approximately 40%, similar to that observed in CF [32]. A recent systematic review indicated that combining inhaled and intravenous therapy leads to higher *P. aeruginosa* clearance rates than intravenous therapy alone [32]. Additionally, an analysis of bronchoalveolar lavage samples from 147 patients with bronchiectasis revealed that 60% of *P. aeruginosa* isolates were resistant to at least one antibiotic group based on the minimal inhibitory concentrations determined by the Clinical and Laboratory Standards Institute [28].

In studies of adults with COPD complicated with *P. aeruginosa* infections, the mortality rate was one to two times higher compared to those without the infection and three times higher in patients with persistent *P. aeruginosa* isolates compared to those with only a single isolation [33,34,35,36].

## 4. Mechanisms and Factors Driving Antibiotic Resistance in *Pseudomonas aeruginosa*

**Figure 1:**
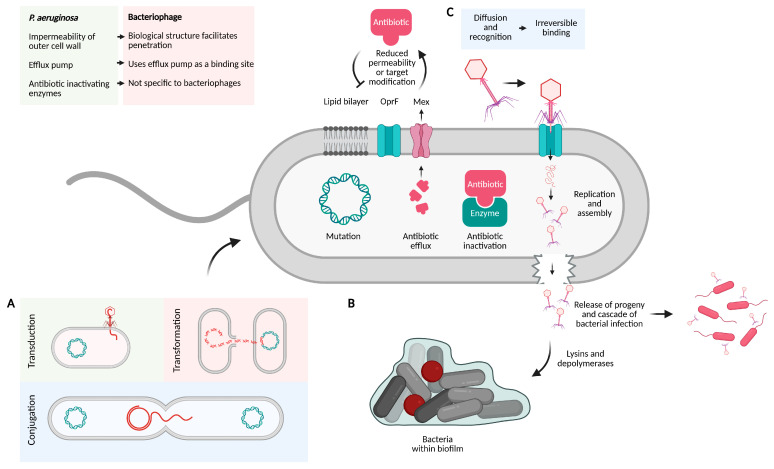
Schematic diagram illustrating the mechanisms of antibiotic resistance in *Pseudomonas aeruginosa* and the ability of bacteriophages to overcome them. Mechanisms of antibiotic resistance in *P. aeruginosa* include impermeability of the outer cell wall membrane (lipid bilayer, restrictive porin channels such as OprF), the presence of efflux pumps (e.g., Mex), production of antibiotic-inactivating enzymes, mutational changes that reduce antibiotic uptake, target modification, and overexpression of efflux pumps or inactivating enzymes. (**A**) illustrates the mechanisms of horizontal gene transfer. (**B**) depicts biofilm formation, highlighting the presence of persister cells (marked in red). (**C**) provides an overview of bacteriophage diffusion, binding to a surface protein of a bacteria (e.g., OprF), replication, progeny release, and further infection of *P. aeruginosa*. OprF: Major outer membrane protein F; Mex: Multidrug efflux system. The legend (top left) summarises the key resistance of *P. aeruginosa* and how bacteriophages can overcome it. Created in BioRender.

*P. aeruginosa* develops resistance to antibiotics through a combination of intrinsic, acquired, and adaptive mechanisms, posing a significant challenge in the management of suppurative lung diseases [4,37,38] (Figure 1).

The intrinsic resistance mechanism of *P. aeruginosa* includes the impermeability of its outer cell wall membrane, the presence of efflux pumps, and its ability to produce antibiotic-inactivating enzymes [4]. The reduced permeability of the outer cell wall, a bilayer structure consisting of phospholipids and lipopolysaccharides and embedded outer membrane proteins, including so-called porins, is due mostly to the dominance of the ‘slow porin’ OprF. OprF are highly restrictive to the uptake of antibiotics and has been shown to contribute to resistance against macrophage clearance [37,39].

Efflux pumps play a critical role in expelling toxic compounds from the bacterial cell. In *P. aeruginosa*, the efflux pumps most relevant to antibiotic resistance belong to the resistance-nodulation-division (RND) family. These protein complexes include cytoplasmic and periplasmic components, collectively referred to as multidrug efflux systems (Mex). Together with restricted porin channels, they are major contributors to antibiotic resistance in the bacterium [40].

Four major efflux pump systems in *P. aeruginosa* contribute to its antibiotic resistance: MexAB-OprM (effective against beta-lactams and quinolones), MexCD-OprJ (effective against beta-lactams), MexEF-OprN (effective against quinolones), and MexXY-OprM (effective against aminoglycosides) [41]. Overexpression and mutations in these efflux pump systems have been observed in CF, indicating a broadening of antibiotic resistance and the development of multidrug-resistant *P. aeruginosa* [42].

*P. aeruginosa* produces antibiotic-inactivating enzymes such as β-lactamases and aminoglycoside-modifying enzymes. These enzymes exploit the susceptibility of chemical bonds such as amides and esters, leading to the hydrolysis of beta-lactam antibiotics and aminoglycosides [43]. More recently, a rise in the production of carbapenemases has been observed, contributing to the rise of carbapenem resistance in *P. aeruginosa* [44].

In addition to its intrinsic resistance, *P. aeruginosa* can develop resistance through adaptive mechanisms, which include mutational changes and horizontal gene transfer [45].

Mutational changes involve genetic alterations that reduce antibiotic uptake, modify antibiotic targets, and lead to the overexpression of efflux pumps or antibiotic-inactivating enzymes. Studies of clinical isolates from CF clinics worldwide have demonstrated significant mutational differences between early and late isolates of *P. aeruginosa*. These include mutations in genes linked to the formation of biofilms (mucA, lgU), a reduction in virulence (ykoM and mpl), and changes in regulatory systems, including quorum sensing (rpoN, lasR) [46]. On average, 0.5 to 12 single-nucleotide polymorphisms (SNPs) and 0.4 to 2.7 insertions or deletions (indels) occur per year [47]. This process is further accelerated in hypermutator strains observed in 30% of CF clinical isolates [48]. These strains typically emerge after five years of infection and significantly increase the likelihood of acquiring advantageous mutations, enhancing the bacterium’s ability to adapt and resist antibiotics [49,50]. In CF, hypermutator strains of *P. aeruginosa* are found in about 1 in 3 patients. These strains contribute to poor patient outcomes due to their ability to rapidly adapt to antibiotic exposure, with a mutation rate 1000 times higher than wild strains of *P. aeruginosa* [51].

Horizontal gene transfer facilitates the spread of antibiotic resistance genes through plasmids, transposons, integrons, and bacteriophages (temperate bacteriophages) between the same or different bacterial species [52]. This process occurs via three primary mechanisms: conjugation, where genes are transferred through direct physical contact between bacterial cells; transduction, where genes are transferred by temperate bacteriophages (bacteriophages that integrate their genetic material into bacterial genomes); and transformation, where bacteria take up free deoxyribonucleic acid (DNA) fragments released into the environment and incorporate them into their own genome [4].

*P. aeruginosa* undergoes adaptive changes to survive in the presence of antibiotics through transient alterations in gene and protein expression, which can be reversed when the stimulus is removed [53]. Biofilm formation is one such adaptive mechanism, resulting from adaptation to the suppurative lung environment. This biofilm, composed primarily of extracellular polymeric substances, predominantly alginate, forms an additional barrier that impedes antibiotic penetration. Furthermore, acetylation of alginate provides additional protection by reducing immune recognition of the bacterium, rendering it resistant to opsonisation [54]. *P. aeruginosa* utilises quorum sensing, an intracellular communication system mediated by small signalling molecules such as N-acyl homoserine lactones. These molecules regulate the expression of bacterial genes and modulate behaviours such as biofilm production, particularly when exposed to antibiotics [55].

Another challenge in treating *P. aeruginosa* infections is the bacterium’s ability to adapt and form persister cells as part of its adaptive strategy for survival. These cells are genetically identical to the rest of the bacterial population but exhibit phenotypic variations due to heterogeneous environmental conditions [56]. Persister cells, which form a subpopulation of biofilm cells, are typically non-growing and metabolically inactive [57]. These cells remain dormant during antibiotic exposure, resume growth, and repopulate biofilms once the antibiotic is removed, making them a key driver of recalcitrant infections [58].

## 5. Bacteriophage Therapy as a Promising Approach to Overcome Difficult-to-Treat *P. aeruginosa* Infections in CSLD

Bacteriophages are ubiquitous and diverse viruses that specialise in infecting bacteria. The term ‘bacteriophage’, derived from Latin meaning ‘bacteria eaters’, refers to their ability to infect and replicate within bacterial cells, ultimately leading to the destruction of the bacterium [59]. While bacteriophages have been identified and used in therapy for over a century in countries like Georgia and Poland, the majority of the world has relied on antibiotics for bacterial infections [60]. Faced with the rise of antimicrobial resistance and the dwindling prospects of new antibiotic discovery, the Western scientific community is increasingly interested in alternatives or complements to antibiotics, particularly bacteriophages [61].

The typical structure of a bacteriophage includes a head, neck, sheath, tail, and baseplate [62]. Bacteriophages initiate infection using their tails to recognise and bind to specific receptors on the bacterial surface, such as lipopolysaccharides (LPS), flagella, or pili [63] Many *P. aeruginosa*-infecting bacteriophages preferentially target type IV pili as an initial binding site, with some also showing an affinity for OmpF receptors [64,65]. Once bound, the bacteriophage employs a series of viral proteins, including tail-associated enzymes (e.g., virion-associated lysins), to locally degrade the bacterial cell wall, facilitating genome injection into the cytoplasm [63,66]. Bacteriophages such as OMKO1 (family *myoviridae*) have also demonstrated an affinity for using efflux pumps, for example, by utilising the outer membrane protein M of the mexAB—and mex-XY-multidrug efflux systems of *P. aeruginosa* as binding sites, thereby forcing the bacterium into an evolutionary trade-off [67,68]. In this process, the evolution towards bacteriophage resistance can alter the efflux pump mechanism, thereby weakening bacterial resistance mechanisms and restoring the effectiveness of antibiotics [69]. Following this, the bacteriophage injects its genome into the cytoplasm of *P. aeruginosa* [70]. Therapeutic bacteriophages are predominantly lytic, meaning they begin replicating immediately after infection, assembling new phage particles and ultimately causing host cell lysis [71]. This process may involve additional phage-produced proteins, such as endolysins, which disrupt the bacterial membrane, leading to the release of bacteriophage progeny [72,73]. 

Bacteria can develop resistance to bacteriophages through various mechanisms, including: surface modifications that prevent bacteriophage adsorption (such as losing a receptor, downregulating its expression, mutating the receptor, or masking it); superinfection exclusion (preventing the entry or replication of the bacteriophage genome within the bacterium); restriction-modification systems (a bacterial immune mechanism that cleaves unmodified foreign DNA while leaving self-modified DNA untouched); the CRISPR-Cas system (an adaptive bacterial immune system that maintains a genetic memory of invaders and produces complexes that bind to and cleave complementary nucleic acids); and abortive infection (an altruistic mechanism where the infected bacterium dies to prevent the development and spread of bacteriophage progeny) [74,75]. However, these changes come at a high physiological cost, diminishing the bacteria’s infectivity by reducing motility and growth rates [76]. Even when bacteriophage-resistant strains develop, they often fail to reproduce in vivo, and clinical outcomes remain favourable despite the emergence of resistance [77,78].

Unlike the bacterium-antibiotic system, the bacterium-bacteriophage system is dynamic and evolving. Bacteriophages are replicating biological entities capable of exponential growth, and they generate their own mutations reciprocally that can effectively target and eliminate emergent bacterial resistance towards the initially administered bacteriophage [79,80].

Antibiotics are designed primarily to act on bacterial cells. They do not readily penetrate and destroy biofilms, requiring a much higher concentration in vivo to penetrate these additional layers into the bacterium. This allows time for the bacterium to adapt to the presence of the antibiotic [81,82]. Bacteriophages and their interaction with bacterial biofilms are achieved through several mechanisms. Bacteriophages can produce or induce the bacterial host to produce enzymes (lysins and depolymerases) that degrade extracellular polymers by breaking down the polysaccharide matrix and proteins within the biofilm, thereby facilitating their entry into the bacteria. Additionally, bacteriophages can penetrate the inner layers by diffusing through biofilm water channels or targeting the appendages of motile bacteria to enable bacteriophage adsorption [83,84].

A recent study on *P. aeruginosa* bacteriophages demonstrated their ability to target and kill dormant, metabolically inactive persister cells. It is postulated that this occurs through two potential mechanisms. First, bacteriophages may subvert the host’s dormant physiology by mobilising stored resources and energy to enable replication. Second, bacteriophages may invade persister cells but remain dormant, postponing replication until conditions improve. In this pseudolysogeny state, the bacteriophages are protected against environmental hazards. Once the infected bacterium exits dormancy and resumes metabolic activity, the bacteriophage transitions to active replication, ultimately leading to the lysis of the host cell. This ensures the completion of the bacteriophage’s lytic cycle and eradication of the persister cells [85].

An important aspect of bacteriophage therapy’s effectiveness lies in its combination with antibiotics, resulting in bacteriophage-antibiotic synergy. Several examples of this synergy have been described. For instance, in developing resistance to bacteriophages, bacteria often lose resistance to antibiotics as an evolutionary trade-off [86]. The combination of bacteriophages and antibiotics enables simultaneous targeting of bacteria at different sites, making it difficult for bacteria to develop resistance due to the diverse attacks on multiple receptors [87]. Certain antibiotics, such as those inhibiting bacterial cell division, cause bacterial cells to elongate and may enhance bacteriophage replication [88,89]. Additionally, antibiotics like ciprofloxacin have been shown to increase bacteriophage replication rates [90]. Bacteriophages also disrupt bacterial biofilms, thereby facilitating deeper penetration of antibiotics into bacterial communities [91].

While the impact of bacteriophages on the human immune system, particularly regarding their pro- and anti-inflammatory effects, remains relatively unclear, they have been shown to induce a proinflammatory response that aids in bacterial clearance. In experiments involving bacteriophage therapy for *P. aeruginosa* infections in laboratory mice, all immunocompetent mice survived. In contrast, neutropenic mice did not, suggesting neutrophils play a crucial role in bacterial clearance facilitated by bacteriophages [92,93]. Bacterial elimination can also occur through the recognition of bacteriophage- and bacterial-derived pathogen-associated molecular patterns (PAMPs), which stimulate the local immune response by activating macrophages to phagocytose bacteriophage-infected bacteria [94,95].

## 6. Bacteriophage Therapy for Chronic Respiratory Infections: Advancements and Clinical Outcomes

Facing MDR *P. aeruginosa* infections, bacteriophage therapy is increasingly recognised as a promising strategy for treating lung infections. Research into bacteriophage therapy currently progresses through two key approaches: compassionate access schemes, which allow treatment for patients with no other therapeutic options across a variety of infections (as determined by a physician) with subsequent outcome monitoring [96,97,98,99], and clinical trials focused on a single disease, featuring standardised administration, dosage, duration and monitoring in line with evidence-based medicine practices [100,101] Both approaches are crucial for advancing our understanding of bacteriophage therapy [102]. A summary of key clinical trials are described in Table 1, and published case reports are described in Table 2.

As described in Table 1 and Table 2, bacteriophage therapy for chronic respiratory infections is primarily delivered via intravenous and nebulised routes, with one clinical trial also utilising bronchoscopic administration alongside nebulisation. The optimum administration route, dosing, and duration of bacteriophage therapy remain unknown and will require further elucidation through thorough investigations of the immunological responses that these methods elicit [103]. Focus on the paediatric population and chronic suppurative lung diseases outside CF remains limited.

**Table 1:** Clinical trials in bacteriophage therapy in cystic fibrosis and non-suppurative chronic lung diseases (both ongoing and completed) registered on clinical trial registries (United States: clinicaltrials.gov, Australia and New Zealand: anzctr.org.au, International: trialsearch.who.int, European Union: clinicaltrialsregister.eu), accessed on 1 January 2025.

Study	Sponsor	Type of Study	Key Criteria	Intervention	Status	Major Outcomes
Bacteriophage Therapy of Difficult-to-treat Infections (BT100) NCT05498363	Queen Astrid Military Hospital 2008	Retrospective, based on compassionate access	Patients with difficult-to-treat infections, including LRTIs.	Suitable bacteriophages were selected from a repository of 25 individual bacteriophages and six bacteriophage cocktails with routes determined by investigators.	Completed with results published [96]	29/114 (25%) of patients within the study treated for LRTI, % of *P. aeruginosa* and condition leading to LRTI unclear. Clinical improvement of at least one symptom associated with the original bacterial infection or presence of an adverse reaction, as assessed by the treating physician, was observed in 77.2% (overall), and microbial eradication was observed in 61.3% (overall).
Phase 1/2 Study Evaluating Safety and Tolerability of Inhaled AP-PA02 in Subjects With Chronic *Pseudomonas aeruginosa* Lung Infections and Cystic Fibrosis (SWARM-Pa) NCT04596319	Armata Pharmaceuticals (Los Angeles, CA, USA) 2020	Phase 1b/2a, double-blind, randomised, placebo-controlled, single and multiple ascending dose study	Cystic fibrosis patients aged 18 years or older with chronic *P. aeruginosa* infection with FEV_1_% > 40%	Nebulised AP-PAO2	Completed with results published in clinicaltrials.gov accessed on 1 January 2025	29 study participants (21 treatment arm vs. eight placebo arm). Adverse events reported in patients within the treatment arm included small bowel obstruction (n = 1), nausea (n = 1), chill (n = 2), chest discomfort (n = 1), fatigue (n = 1), pain (n = 1), pyrexia (n = 1), infective exacerbation of CF (n = 1), sialadenitis (n = 1), vulvovaginal candidiasis (n = 1), injury or procedural complications (n = 2), hypoglycemia (n = 1). Headache (n = 1), trigeminal neuralgia (n = 1)
CYstic Fibrosis bacterioPHage Study at Yale (CYPHY) NCT04684641	Yale University 2021	Randomised, placebo-controlled, double-blinded to evaluate the efficacy and safety of YPT-01	Cystic fibrosis patients aged 18 years or older with at least one occasion of *P. aeruginosa* within the past 2 years and at the screening visit, an FEV_1_% ≥ 40%, clinically stable lung disease.	Nebulised YPT-01 3 mL daily for 7 days	Completed with results published in clinicaltrials.gov and early data presented at a conference [104]	Eight study participants (four in the treatment arm vs. four in the placebo arm) A reduction in *P. aeruginosa* CFU/mL of −0.59 CFU/mL in the treatment arm vs. −0.89 in the placebo arm, infective exacerbation of CF (n = 1) was observed in the treatment arm.
Bacteriophage Therapy for Difficult-to-treat Infections: the Implementation of a Multidisciplinary Phage Task Force (PHAGEFORCE), NCT06368388	Universitaire Ziekenhuizen KU Leuven 2021	Observational based on compassionate access	Participants must be diagnosed with a musculoskeletal infection, chronic rhinosinusitis, sepsis, lung infection (such as cystic fibrosis or bronchiectasis), or hidradenitis suppurativa, and must have failed all previous treatments, including surgical and antibiotic interventions, or have no other available treatment options.	Nebulised or as determined by the task force	Recruiting	N/A
Standardised Treatment and Monitoring Protocol to assess safety and tolerability of bacteriophage therapy for adult and paediatric patients (STAMP study) ACTRN12621001526864	Westmead Institute for Medical Research 2021	Observational based on compassionate access	Patients for whom at least two suitably qualified clinical specialists have agreed phage therapy should be used in difficult-to-treat infections, including lung infections	Route determined as per the investigators	Recruiting	N/A
A Phase 1b/2 Trial of the Safety and Microbiological Activity of Bacteriophage Therapy in Cystic Fibrosis Subjects Colonized With *Pseudomonas aeruginosa*, NCT05453578	National Institute of Allergy and Infectious Diseases (NIAID) 2022	Phase 1b/2, multi-centred, randomized, double-blind, placebo-controlled trial	Cystic fibrosis patients aged 18 years or older with at least one occasion of *P. aeruginosa* within the past 12 months	Intravenous administration with ascending doses arms	Recruiting	N/A
A Single-Arm, Open-Labelled, Safety and Tolerability of Intra-bronchial and Nebulised Bacteriophage Treatment in Children with *Pseudomonas aeruginosa* (CHIP-CF) ACTRN12622000767707	The Children’s Hospital at Westmead, Sydney Children’s Hospital Network 2022	Single-Arm, Open-Labelled, Safety and Tolerability Study	Children and adolescents from 6 to 18 years with chronic suppurative lung disease, chronic *P. aeruginosa* infection (>50% of airway samples over 12 months)	Bronchoscopic (Day 1) and nebulised bacteriophage for (Day 2 to 7), 10^8^ PFU/4 mL of suitable bacteriophage determined by investigators	Recruiting with early data published [105].	Two patients were treated with good tolerability and safety (absence of temporal fever, bronchospasm) and eradication of *P. aeruginosa* in 1 patient.
Study to Evaluate the Safety, Phage Kinetics, and Efficacy of Inhaled AP-PA02 in Subjects with Non-Cystic Fibrosis Bronchiectasis and Chronic Pulmonary *Pseudomonas aeruginosa* Infection (Tailwind) NCT05616221	Armata Pharmaceuticals, Inc., (Los Angeles, CA, USA) 2023	Phase 2, multi-centre, double-blind, randomized, placebo-controlled study to evaluate the safety, phage kinetics, and efficacy of inhaled AP-PA02	Patients over 18 years with evidence of bronchiectasis on CT and chronic *P. aeruginosa* infection	Nebulised AP-PAO2	Completed	N/A
PHAGEinLYON Clinic Cohort Study: A Descriptive Study of Severe Infections Treated with Phage Therapy at the Hospices Civils de Lyon NCT06185920	Hospices Civils de Lyon 2023	Non-interventional retrospective and prospective study based on compassionate access	Patients with severe infections potentially including lung infections treated with bacteriophage in the Hospices Civils de Lyon	N/A	Recruiting	N/A

**Table 2:** Published case reports on bacteriophage therapy used to treat *P. aeruginosa* infection in conditions related to suppurative lung diseases.

Author	Subject	Route and Dosage	Frequency of Administration	Major Outcome Reported
N. Law 2019 [106]	26-year-old female with pulmonary exacerbation of CF	IV cocktail of four lytic bacteriophages AB-PA01 4 × 10^9^ PFU/mL in 5 mL with concomitant IV antibiotics	Six hourly over eight weeks	Improvement in oxygen requirement, fever profile, reduced sputum production and improvement in mobility. Improvement in white blood cell count and resolution of acute kidney injury. No pulmonary exacerbation of CF within 100 days following bacteriophage therapy No regrowth of *P. aeruginosa* within the sputum sample collected
T. Köhler 2023 [107]	41-year-old male with Kartagener syndrome and traumatic spinal injury with tetraplegia with severe lower lobe consolidations	Nebulised single-strain bacteriophage vFB297 5 × 10^9^ PFU/mL with concomitant IV antibiotics	Daily for five days, followed by two additional doses two days later	Progressive clearance of left lower lobe consolidation A reduction of CFU of *P. aeruginosa* was observed
A. Hahn 2023 [108]	6 and 26-year-olds with pulmonary exacerbation of CF	Nebulised single-strain bacteriophage INF 1 × 10^10^ PFU/mL with concomitant IV antibiotics	6-year-old: twice a day for seven days. 26-year-old: twice a day for 2 days of bacteriophage INF followed by once a day for seven days	Both patients demonstrated improvement in terms of oxygen requirement, reduced sputum production and improvement in energy levels In the 6-year-old, *P. aeruginosa* was temporarily not isolated from the sputum culture during treatment. Subsequent *P. aeruginosa* from both patients demonstrated improved susceptibility against antibiotics.
L. Li 2023 [109]	40-year-old man with interstitial lung disease and acute on chronic pulmonary exacerbation	Nebulised single-strain bacteriophage phiYY 10^8^ PFU/mL	Twice at a 4-hourly interval, repeated four days later.	A reduction in sputum production occurred during therapy. *P. aeruginosa* was transiently not isolated from sputum culture during the first course of treatment. Subsequent *P. aeruginosa* isolates demonstrated improved susceptibility against antibiotics

Most compassionate access studies and clinical trials employ cocktail preparations. However, preliminary findings suggest no significant difference in effectiveness between monovalent bacteriophage preparations and cocktail preparations [102]. Studies highlight the distinct advantages of each approach: cocktail preparations can leverage the synergy between bacteriophages, such as biofilm degradation by depolymerase(s) from one bacteriophage, enabling adsorption by another, delaying the development of bacterial resistance to an individual or all bacteriophages in the cocktail, and targeting multiple bacterial species or strains. Bacteriophage cocktails can be designed to offer both depth and breadth of therapeutic efficacy, tailored to the specific needs of the condition. This broader therapeutic spectrum is especially valuable in treating bacterial infections caused by multiple resistant strains [110]. Conversely, monovalent bacteriophage preparations reduce the potential for cross-resistance between bacteriophages and may elicit a lower antibody response [102,110,111].

While there are slight variations in the selection and production of bacteriophages for therapy, a few core principles are common in developing and delivering bacterio-phage treatment [112]. The first step is to identify a suitable bacteriophage active against the target bacteria. This is determined using bacterial isolates from patients, which are tested against various bacteriophages using a combination of in vitro techniques. These include spot tests (applying phage droplets onto a bacterial lawn to observe zones of inhibition or clearance), plaque assays (serial dilution of phage samples to produce countable individual plaques), and growth kinetics assays (monitoring bacterial growth inhibition in real-time via optical density measurements) [112,113].

Once suitable bacteriophage(s) are selected, they are propagated using a bacterial host to increase titres to the concentration required for treatment. Purification of the phage preparation is a critical step, aimed at removing contaminants that may provoke an inflammatory response in the human host. These contaminants include endotoxins, bacterial nucleic acids, host cell proteins, and media components used during production. In the absence of phage-specific regulatory guidelines, most bacteriophage producers develop their own internal quality control processes and protocols [114,115,116].

In terms of outcomes, several studies have reported positive clinical results, including favourable patient outcomes, the absence of adverse events, and either a reduction or complete eradication of the target organism, as summarised in Table 1.

## 7. From Compassionate Access to Routine Care: The Next Steps for Bacteriophage Therapy

The major hurdle in advancing bacteriophage therapy lies in the characteristics of bacteriophages themselves [117,118]. While advantageous for targeted treatment, their diversity and narrow strain specificity make standardisation, manufacturing, and regulation particularly challenging. Identifying a suitable bacteriophage for a given infection requires timely microbiological diagnosis and access to an appropriate bacteriophage library—resources that are not yet universally available [119].

The diversity and specificity of bacteriophages make standardisation and regulation challenging. To address this, several countries have established alternative regulatory pathways for accessing bacteriophage therapy through compassionate use schemes, which have shown promising results in diverse patient groups. In Australia, bacteriophage therapy is available through the Special Access Scheme (SAS), allowing infectious disease specialists to prescribe it, or via the Therapeutic Goods Administration’s Clinical Trial Notification or Clinical Trial Exemption pathways [97,101]. In Europe, the European Medicines Agency (EMA) has classified bacteriophages as medicinal products since 2011, and the Bacteriophage Working Party was recently created to support their regulation. In the United Kingdom, bacteriophages used for therapy must adhere to Good Manufacturing Practice (GMP), and those produced locally for clinical trials are subject to GMP production regulations [120]. In the United States, bacteriophages are classified as biological products and must meet GMP standards, provide preclinical data, and undergo clinical trials [121]. In exceptional cases, an Investigational New Drug (IND) application can be submitted to the FDA, similar to the Australian SAS pathway [122]. While countries are moving towards standardising clinical trial and therapy approaches, well-designed trials are urgently needed to provide regulators with comprehensive safety and efficacy data. Currently, most trials rely on compassionate use pathways, resulting in significant heterogeneity in treatment duration, underlying conditions, delivery methods, dosing, selection criteria and the subsequent analysis and interpretation of results. Though these trials provide valuable data, planning for multi-centred, multinational phase 2 trials are essential. Such studies must establish standardised procedures, dosing strategies, and clinical endpoints to generate robust evidence for bacteriophage therapy’s safe and effective integration into routine clinical practice.

Further limitations of bacteriophage therapy include its impact on immune responses, such as the development of neutralising antibodies, which may reduce therapeutic efficacy, especially with repeated or prolonged use [123,124,125,126]. The role of bacteriophages in eliciting an immunogenic response should be carefully considered, and methods that minimise the immune response should be prioritised [119]. Ongoing studies aim to provide further insights into how the dose and route of delivery influence the immunogenic response to bacteriophages [97,101]. Several strategies have been proposed to address the emergence of bacteriophage resistance. These include the concurrent use of bacteriophage therapy with antibiotics, thereby leveraging the potential synergism between the bacteriophage and antibiotic against the bacterial host. As discussed earlier, bacteriophage cocktails that broaden the host range are another commonly used strategy. Table 3 compares the advantages and limitations of antibiotics against bacteriophage therapy.

## 8. Conclusions and Future Directions

The importance of clinical trials in bacteriophage therapy is particularly important for conditions such as chronic suppurative lung diseases, where treatment for MDR bacteria has often been unsuccessful. Although these infections are typically chronic and indolent, they require bacterial eradication to prevent long-term deterioration. Compassionate access schemes, while valuable, may not always focus on such cases unless the patient is critically unwell, underscoring the need for dedicated research to address the unique challenges of these conditions. Furthermore, the development of bronchiectasis following a prolonged infection could be mitigated by early eradication of these organisms as soon as conventional treatment is determined to have failed. This is particularly crucial in children with chronic suppurative lung disease, where the risk of bronchiectasis significantly impacts their well-being and long-term health outcomes.

To conclude, clinicians and academics in the growing bacteriophage field must urgently collaborate to conduct priority-setting exercises. These efforts are essential to defining clear research goals, streamlining development pathways, and charting a focused and actionable roadmap for the future of bacteriophage therapy in the lungs, ensuring its timely translation into routine clinical practice.

## Figures and Tables

**Table 3:** Comparative Overview of Antibiotic and Bacteriophage Therapy for Chronic Respiratory Infections. (Readers are encouraged to consider their synergistic potential, as discussed in the text).

Aspect	Antibiotic Therapy	Bacteriophage Therapy
*Clinical Evidence*	Supported by large-scale, late-phase clinical trials and decades of post-marketing surveillance.	Growing evidence from preclinical studies, early-phase clinical trials, and compassionate access programmes.
*Effectiveness*	Standard of care for many decades to treat infections.	Emerging evidence demonstrates effectiveness against multidrug-resistant organisms.
*Route and Dosing*	Standardised dosing with established routes of administration and well-defined regimens for most infections.	Optimal dosing and route of administration remain unclear and may depend on the specific bacteriophage used and the clinical context.
*Safety*	Both short- and long-term safety profiles are well documented, including the potential for allergic reactions, gastrointestinal disturbances, and end-organ toxicity.	Recent studies suggest that bacteriophages are generally well-tolerated. However, the long-term safety profile remains under investigation.
*Complications*	It may cause antibiotic-associated diarrhoea, secondary infections, or end-organ damage. Drug interactions may complicate use.	It may induce immune or inflammatory responses, failure to identify a suitable bacteriophage, or insufficient potency against the target strain.
*Impact on Microbiome*	Broad-spectrum antibiotics disrupt gut and respiratory microbiota, leading to secondary infections or long-term alterations.	Typically, more specific, with less disruption to the microbiome, although long-term effects are not yet fully understood.
*Development of Resistance*	The overuse or misuse of antibiotics can lead to a high potential for resistance development, a major global health concern.	There is a lower risk of resistance due to high specificity, although resistance to bacteriophages can still occur.
*Efficacy in the Paediatric Population*	Extensive data support the use of antibiotics in children.	There is very limited data in children, particularly those with chronic respiratory diseases outside of CF.
*Regulatory Hurdles*	Well-regulated with established pipelines for approval, distribution, and clinical use.	Regulatory frameworks are less developed, vary between countries, and can be complex, limiting broader implementation.
*Cost*	Generally, low production and distribution costs, though development can be expensive.	High costs due to individualised production, testing, and regulatory hurdles. Economies of scale are currently limited.
